# Electrophysiological characterization of neuropeptide S-expressing neurons in the periLC: influence of the female estrous cycle on neuronal excitability in mice

**DOI:** 10.1007/s00424-026-03192-x

**Published:** 2026-07-18

**Authors:** Nike Leah Laukamp, Kay Jüngling

**Affiliations:** Institute of Physiology I, Medical Faculty of the University Münster, Robert-Koch Str.27a, 48149 Münster, Germany

**Keywords:** Neuropeptide S, Estrous cycle, Potassium current, Sex-dependent differences, Electrophysiology, Peri-coerulear region

## Abstract

Neuropeptide S (NPS) signaling is critically involved in arousal, sleep–wake regulation, fear and anxiety, and related forms of learning and memory, yet comparatively little is known about whether intrinsic properties of identified NPS neurons differ between sexes or fluctuate across the female estrous cycle. Here, we performed whole-cell patch-clamp recordings from genetically identified NPS-eGFP neurons in acute horizontal brainstem slices containing the peri–locus coeruleus (periLC) region. We show that periLC NPS neurons can be subdivided into two electrophysiologically distinct classes based on their discharge patterns from hyperpolarized membrane potentials: a delayed-onset firing phenotype (type I) and a regular-spiking phenotype (type II). Pharmacological and voltage-clamp analyses indicate that differential contribution of a transient A-type potassium current (I_A_), consistent with Kv4 channel function, is a key determinant of these firing phenotypes and provides a mechanism for gain control in type I neurons. Across male and female mice, we did not detect robust sex differences in the relative abundance of these neuronal classes or in overall excitability measures, suggesting that baseline intrinsic properties of periLC NPS neurons are broadly comparable between sexes. In contrast, estrous staging revealed a selective modulation of intrinsic discharge in females: during non-receptive stages (metestrus/diestrus), type I neurons exhibited higher spike output and increased instantaneous firing frequencies compared with receptive stages (proestrus/estrus), whereas type II neurons were comparatively stable across the cycle. These findings provide a cellular mechanism by which ovarian state may tune NPS neuron output and, consequently, the impact of NPS signaling on downstream circuits controlling arousal and stress-related behaviors.

## Introduction

The neuropeptide S (NPS) transmitter system consists of the 20 amino acid neuropeptide S and its G protein-coupled receptor, NPSR. Experimental activation of NPSR by systemic or central NPS administration has been shown to affect food-intake [35], the sleep–wake cycle and states of arousal [40–42], general anxiety [12, 24, 32, 42], and extinction of conditioned fear responses in vivo [3, 12, 27]. There is good evidence that the NPS system is critically involved in learning and memory of fear-related and other behaviors in different animal models. Furthermore, human studies demonstrate that polymorphisms of NPSR are linked to the interpretation of personal fear reactions and to panic disorders, both of which are associated with alterations in PFC activity [25], [9], [10], [30].

Sex as a biological variable has received comparatively little attention in the preclinical NPS literature. However, the available evidence indicates that NPS/NPSR signaling can differ between males and females and may be modulated across the estrous cycle. In female mice, central NPS/NPSR activation produced anxiolytic-like effects that depended on estrous stage, with stronger behavioral responses during high-estrogen phases [8]. Consistent with this notion, genetic disruption of *Npsr1* revealed sex-specific stress-related phenotypes: enhanced depression-like behavior and altered stress responsiveness were more pronounced in male mice than female mice [43], and *Npsr1* deficiency prevented stress-facilitated safety learning predominantly in males [20]. In addition, sex-dependent outcomes have been reported for social behavior and fear extinction in *Npsr1*-deficient mice [19] and in mice carrying human NPSR1 variants [3, 7, 36]. In rats, NPS similarly shows sex-dependent effects on anxiety- and fear-related behaviors and alcohol seeking [39], [21]. Despite these behavioral and genetic data, direct evidence that the female estrous cycle impacts intrinsic properties of identified NPS neurons is still missing.

In the mouse brain stem, NPS-expressing neurons are located in two spatially distinct regions. One population is positioned between the locus coeruleus (LC) and Barrington’s nucleus (BN), close to the fourth ventricle, whereas the second population is located between the lateral parabrachial nucleus (LPB) and the Kölliker-Fuse nucleus (KF) [42]. Using an NPS-eGFP transgenic mouse line [23], we identified two electrophysiologically distinguishable NPS neuron subpopulations in the periLC region, namely type I delayed and type II regular firing neurons, in both male and female mice. Of note, we provide first evidence for enhanced neuronal excitability predominantly in type I neurons during the non-receptive stage of the female cycle. These findings provide a mechanistic entry point for understanding how ovarian state can tune the NPS system, because changes in intrinsic excitability are expected to impact action potential output and, consequently, NPS release. Together, our data suggest that cycle-dependent shifts in periLC NPS neuron excitability may contribute to sex- and state-specific variability in arousal, anxiety, and fear-related behaviors.

## Methods

### Animals

Transgenic NPS-eGFP mice (transgenic NPS-eGFP mouse line E16; [23] were kept in individually ventilated cages (21 °C,50%–60% relative humidity) with access to food and water ad libitum and a 12-h light/dark cycle with lights on at 6:00 AM. All animal experiments were carried out in accordance with European regulations on animal experimentation (European Committee Council Directive 2010/63/EU; National Research Council of the National Academies), approved by the local authorities (LAVE [State Agency for Nature, Environment and Consumer Protection and Nutrition North Rhine-Westphalia]).

### Electrophysiological recordings *in vitro*

Eight- to ten-week-old male and female mice were used. Horizontal slices (300 µm thick) containing the LC were prepared. We used a Zeiss Axioskope 2 FS plus (with 5 × and 40 × objectives to find periLC and patch cells). Whole-cell patch-clamp recordings (in voltage- or current-clamp mode) were performed as described previously [12]. Briefly, we used patch pipettes made of borosilicate glass (GC150T-10, Harvard Apparatus, Edenbridge, UK), pulled to resistances of 2.5–3 MΩ using a vertical puller (PA-10, E.S.F. Electronic, Göttingen, Germany). Neurons with a resting membrane potential more positive than −55 mV were rejected from analysis. The intracellular solution contained [in mM]: NaCl 10, K-gluconate 105, K_3_-citrate 20, HEPES 10, BAPTA 3, MgCl2 1, MgATP 3, and NaGTP 0.5. The pH was adjusted to 7.25. Artificial cerebrospinal fluid (ACSF) was used as extracellular solution and contained [in mM]: NaCl 120, KCl 2.5, NaH2PO4 1.25, MgSO_4_ 2, CaCl_2_ 2, NaHCO_3_ 22 and glucose 25. The pH was adjusted to 7.3 by gassing with carbogen (95% O_2_, 5% CO_2_). The liquid-junction potential was corrected for (+ 10 mV). All experiments were performed at 30 °C. In some experiments, tetrodotoxin (TTX, 1 µM) was added to decrease network activity (toxins were purchased from Biozol Diagnostica Vertrieb GmbH, Germany). 4-aminopyridine (4-AP, 10 mM) was bath-applied to block potassium conductances in some experiments. A-currents were isolated using 0.5 µM TTX, 150 µM CdCl_2_ and 10 mM TEA in the ACSF. Of note, whole-cell dialysis may alter intracellular signaling, but all groups were recorded under identical conditions, so the relative group differences remain interpretable.

Electrophysiological data were acquired with an EPC10-double amplifier (HEKA, Germany) at a sampling rate of 10 kHz. Electrophysiological recordings were analyzed using Clampfit 10 (Axon Instruments, Molecular Devices) and subsequently exported to Origin 9.5 for statistical analysis and graphical presentation. Recordings were excluded if any of the following criteria were met: (i) series resistance (R_s_) changed by > 20% during the experiment or increased above 20 MΩ, (ii) resting membrane potential was more depolarized than − 55 mV, or (iii) values were identified as outliers by Grubbs’ test.

Passive membrane properties were quantified in whole-cell configuration. Resting membrane potential was measured immediately after membrane disruption. A hyperpolarizing current step (− 100 pA) was used to determine input resistance (R_in_), membrane time constant (τ), and membrane capacitance (C). R_in_ was calculated as R_in_ = ΔV/I, where ΔV is the steady-state voltage deflection and I is the injected current. The membrane time constant was obtained by exponential fitting of the voltage response to the − 100 pA step, and capacitance was calculated as C = τ/R_in_.

Action potential (AP) firing was assessed during depolarizing current injections from 0 to + 240 pA. When cells were held at a nominal membrane potential of − 80 mV, two distinct firing phenotypes were observed. Type I (“delayed-onset”) neurons generated an initial AP followed by a pronounced pause and subsequently a sequence of APs, whereas type II (“regular”) neurons lacked this delayed-onset pattern and instead showed progressive spike-frequency accommodation over the train. To classify cells, the first sweep containing ≥ 5 APs was used to compute interspike frequencies between AP1–AP2 (f1), AP3–AP4 (f2), and the last two APs (f3). The ratio f3/f1 (“frequency adaptation index”) was then used for assignment: values > 1 were characteristic of type I neurons, whereas values ≤ 1 were characteristic of type II neurons.

AP waveform parameters were measured from the first AP. AP amplitude was defined as the voltage difference between threshold and peak depolarization. AP half-width was measured at half-maximal amplitude relative to threshold. Afterhyperpolarization (AHP) amplitude was quantified as the most negative membrane potential following the spike relative to AP threshold. The kinetics of the AHP was further subdivided into fast afterhyperpolarization (fAHP) and medium afterhyperpolarization (mAHP), representing the immediate, brief post-spike hyperpolarization and the subsequent hyperpolarization.

Spontaneous synaptic transmission was recorded in the whole-cell voltage-clamp mode using a potassium-gluconate-based intracellular solution. Excitatory postsynaptic currents (sEPSCs) were recorded at −65 mV and spontaneous inhibitory postsynaptic currents were recorded at −5 mV. Recorded synaptic events were analyzed offline using Clampfit 10.

For the comparison of neuronal properties of NPS neurons derived from different sexes, we recorded from 20 males and 27 female mice.

To assess to effect of the corticotropin receptor 1 (CRF1) activation, the CRF1-agonist stressin I (Tocris, UK) was bath applied at a concentration of 500 nM for two minutes at a speed of 3.6 ml/min at a membrane potential of −60 mV in the whole-cell current-clamp mode.

### Female estrous cycle and vaginal cytology

The murine estrous cycle typically lasts ~ 4–6 days and is commonly divided into four stages (proestrus, estrus, metestrus, diestrus). In the present study, cycle stage was determined post mortem by vaginal cytology. Immediately after decapitation, a vaginal smear was collected using a sterile swab moistened with room-temperature water, gently rotated against the vaginal wall, and transferred onto a glass slide. Cells were stained for 5 min with cresyl violet acetate, rinsed with distilled water, and evaluated by light microscopy (Zeiss Axioscope 2 FS plus) using the Jackson Laboratory “Mouse estrous cycle stage identification tool” as a visual reference [5]. Staging was based on the relative abundance of leukocytes, nucleated epithelial cells, and cornified epithelial cells, with leukocytes being scarce in proestrus/estrus but prominent in metestrus/diestrus [5]. For statistical analyses, the four stages were pooled into two groups: proestrus/estrus (PE) and metestrus/diestrus (MD). This grouping was chosen to ensure adequate sample sizes and to reduce ambiguity at stage transitions, where cytological classification can be experimenter-dependent. Moreover, this dichotomization captures major differences in reproductive receptivity and aligns with the overall hormonal milieu across the cycle [33], and has been successfully applied in prior work [6].

### Statistics

Data are represented as boxplots, in which the box represents the first (25%) and third (75%) quartiles, the band represents the median, and the whiskers represent the 5th and 95th percentiles. The square within the box represents the mean. Repeated measures, 1-way, or multifactorial analysis of variance (ANOVA) tests were used for between- or within-group comparison, as indicated in the Results. Two independent datasets were compared using Student’s t test or the Mann–Whitney U test. Statistical tests were 2-sided. Mean values in time courses are represented with the SEM.

## Results

### Differential discharge patterns of NPS neuronal subpopulations in the periLC

**Fig. 1:**
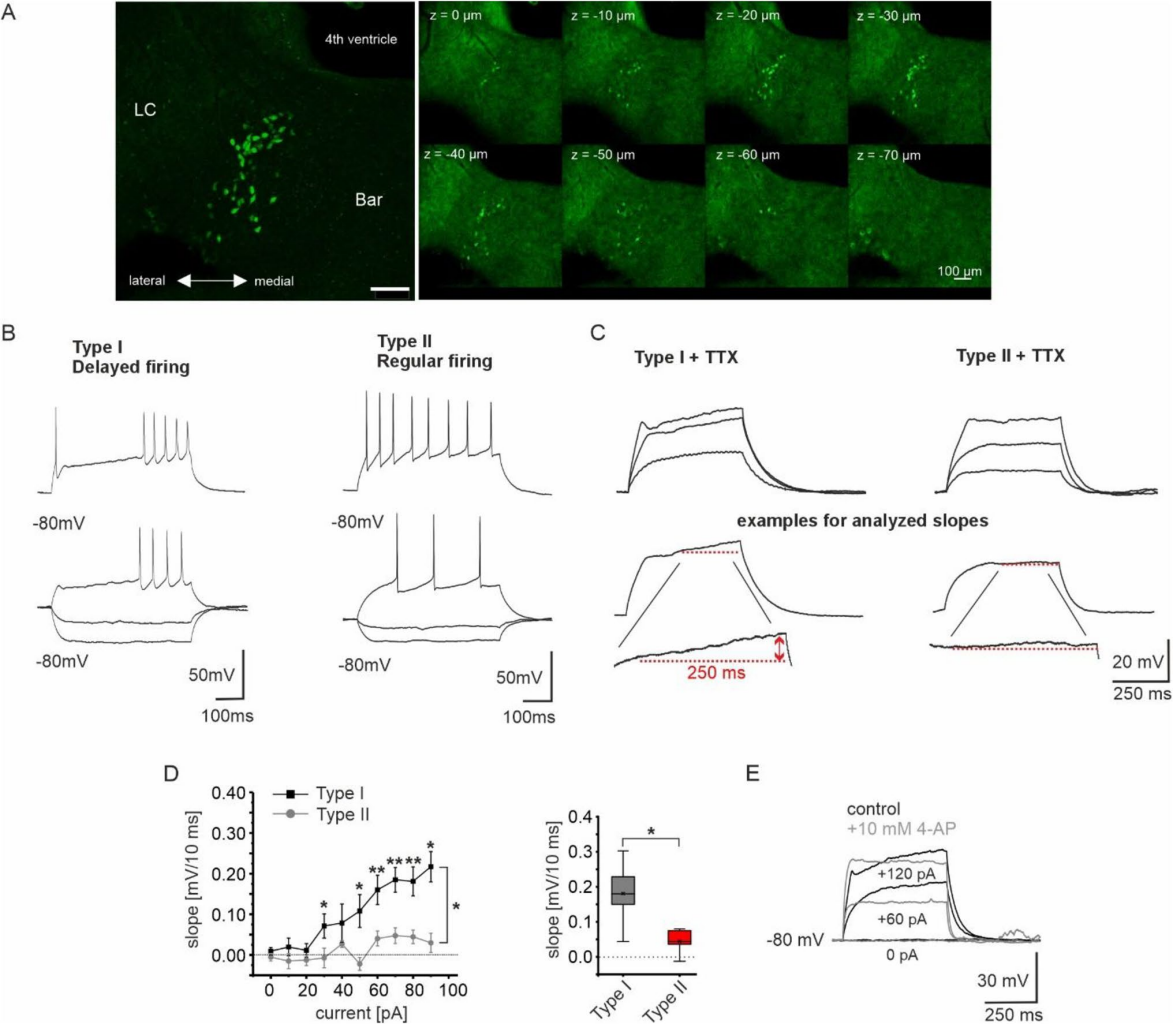
The cluster of NPS expressing neurons in the periLC region consists of two electrophysiologically distinct classes. **A**) Localization of NPS-eGFP neuron periLC cluster in a 500 µm horizontal slice preparation. The fixed slice has been optically cleared using the uDISCO technique. **B**) Discharge pattern of delayed firing (type I) and regular firing (type II) NPS neurons. Hyper- and depolarizing current injections were applied from a membrane potential of −80 mV. **C**) Same current injections in presence of TTX to inhibit voltage-gated sodium channels. Type I neurons show a prominent slope upon depolarizing current injections, whereas type II neurons rapidly reach a steady state. **D**) Quantification of the voltage slope in response to increasing current injections recorded from type I and type II neurons (RM ANOVA with Fishers post hoc test; p-value: *: 0.05; **: 0.001) and statistical comparison of maximal slopes in type I and type II neurons (U-test). **E**) Current clamp recording from a type I NPS neuron in absence and presence (grey) of 10 mM 4-aminopyridine (4-AP)

NPS-eGFP neurons in acute horizontal brain slices were identified using a 450 nm UHPmic LED (Prizmatix) and a GFP filter set. Clusters of NPS neurons are located in the periLC region close to the 4th ventricle (Fig. 1A). NPS neurons in the periLC appear in a band-like structure in the rostro-caudal axis as seen in the stack of optically cleared (uDISCO; [26] 500 µm thick horizontal brain slice (Fig. 1A). We have described basic electrophysiological properties of NPS neurons in the periLC previously [11, 13]. Here we show that NPS neurons in the periLC can be subdivided into two distinct electrophysiological classes. We recorded in the whole cell patch-clamp mode from identified NPS neurons. In the current clamp mode, the membrane potential of these neurons was set to about −80 mV by constant current injections. In response to depolarizing current injections, NPS neurons displayed either a delayed firing response (termed type I) or a regular action potential generation pattern (type II neurons). In the presence of TTX, the membrane potential deflection in response to 500 ms long depolarizing current injections (0 to 90 pA) shows a continuous ramp-like increase not reaching a steady-state in type I neurons. Type II neurons reach a steady-state during depolarizing current injections (Fig. 1C). Analysis of the underlying slope reveals significant differences between type I and type II neurons, where type I neurons show an increase in the slope (mV/ms) of depolarization (RM ANOVA: significant interaction cell type x current injection, F(9,27) = 2.3, *p* = 0.045 type I *n* = 6, type II *n* = 5; Fig. 1D). The peak slope values in response to 90 pA current injections were significantly different between both NPS cell types (U-test: U = 27.5, z = 2.2, *p* = 0.028; type I *n* = 6, type II *n* = 5; Fig. 1D). The observed slope in type I NPS neurons could be pharmacologically blocked by bath application of 10 mM 4-aminopyridine (4-AP; [14], indicating the presence of K_v_ potassium channels mediating a transient A-type current (Fig. 1E).

**Fig. 2:**
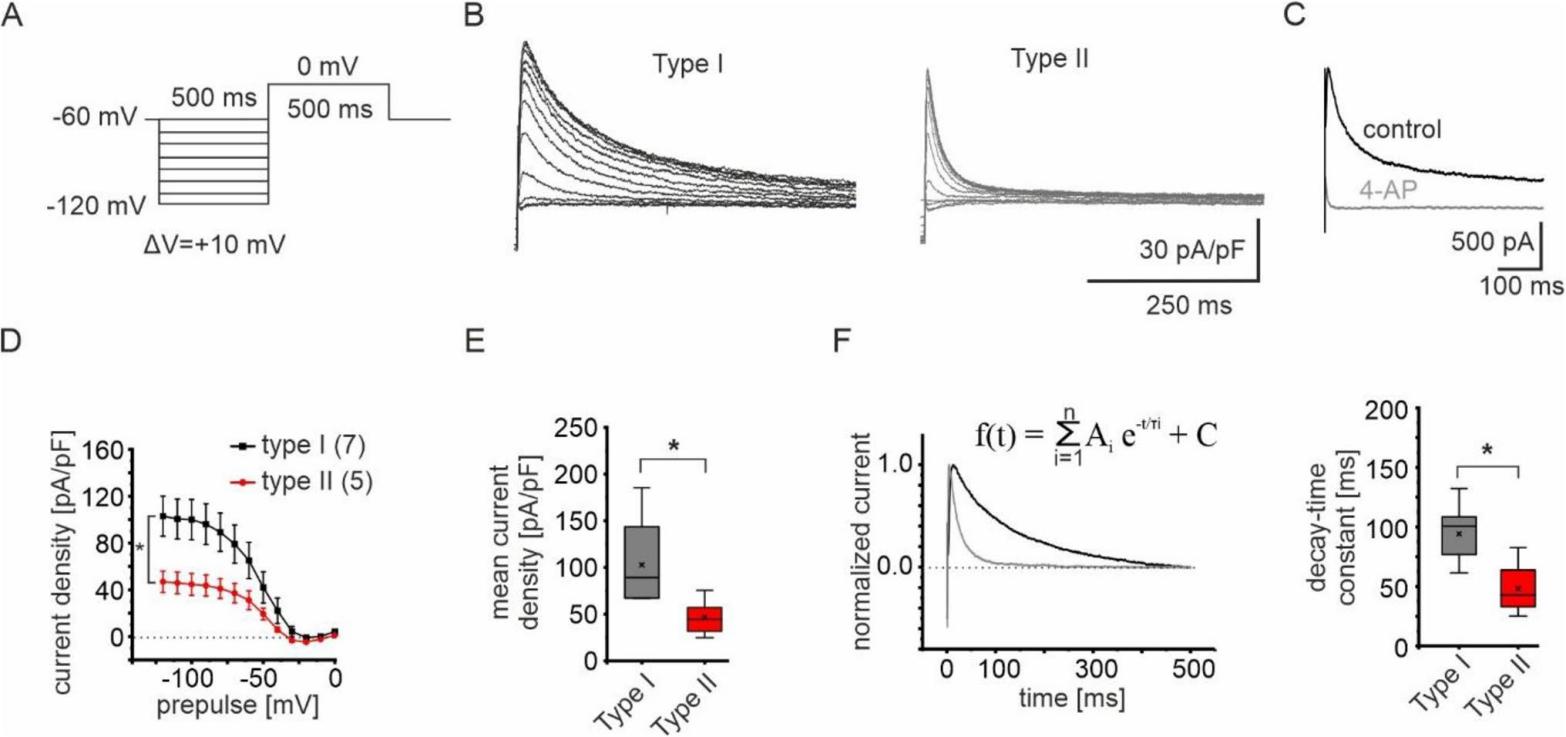
Characterization of transient, voltage-gated outward K⁺ current (I_A_) in type I and II NPS neurons in the periLC region of mice. **A**) Scheme of the used voltage clamp experiment. Pre-pulses of 500 ms duration were used to recover Kv4 channels from inactivation, followed by a voltage step to 0 mV membrane potential to activate the channels. **B**) Example recordings of K_v_4-mediated I_A_ currents from type I and type II neurons. **C**) Application of 10 mM 4-AP abolishes the transient outward-directed current completely in type I neurons. **D**) Inactivation curves of NPS type I and type II neurons differ significantly. **E**) Quantification of the maximal current density recorded from type I and type II neurons following a pre-pulse to −120 mV. **F**) Comparison and quantification of the I_A_ decay slope in both neuronal typed by monoexponential fit

To test the involvement of I_A_-currents directly, in the next set of experiments we recorded I_A_-currents in type I and type II neurons. The recordings were performed in presence of 0.5 µM TTX, 150 µM CdCl_2_ and 10 mM TEA, and type I and type II neurons were distinguished by presence or absence of a depolarization slope in response to depolarizing current injections. I_A_-currents were recorded in voltage clamp at a holding potential of −60 mV. Pre-pulses of 500 ms duration were applied from −120 to −60 mV (ΔV = + 10 mV), followed by a voltage jump to 0 mV to elicit the I_A_ (Fig. 2A) in type I and type II neurons (Fig. 2B). The elicited I_A_ was completely abolished by application of 10 mM 4-AP (Fig. 2C). We analyzed the current density of the elicited I_A_ as a function of conditioning pre-pulse voltage. The derived inactivation curve (Fig. 2D) was significantly different between type I and type II neurons, as expected due to the presence of K_v_4 channels (RM ANOVA: interaction pre-pulse x neuronal type F(12,60) = 2.11, *p* = 0.029). The maximal current density evoked from a pre-pulse of −120 mV was 103 ± 17 and 48 ± 9 pA/pF in type I and type II neurons, respectively (U-Test: U = 32, z = 2.27, *p* = 0.023; Fig. 2E). To identify possible changes of I_A_-current kinetics, we compared the decay-time constant τ derived from the mono-exponential fit of the normalized maximal evoked current (Fig. 2F). The time constant measured in type I neurons was at 94.2 ± 9.0 ms and was significantly larger than the time constant derived from type II neurons with 48.0 ± 12.2 ms (U-test: U = 25, z = 1.98, *p* = 0.047). These findings indicate that NPS type I neurons in the periLC region show a larger and slower K_v_4-like current, which mechanistically underlies the transient outward-directed potassium current I_A_. Moreover, differences in current kinetics might point towards a differential expression of isoforms and/or auxiliary subunits.

### Recordings of type I and type II neurons in the periLC from male and female mice

**Fig. 3:**
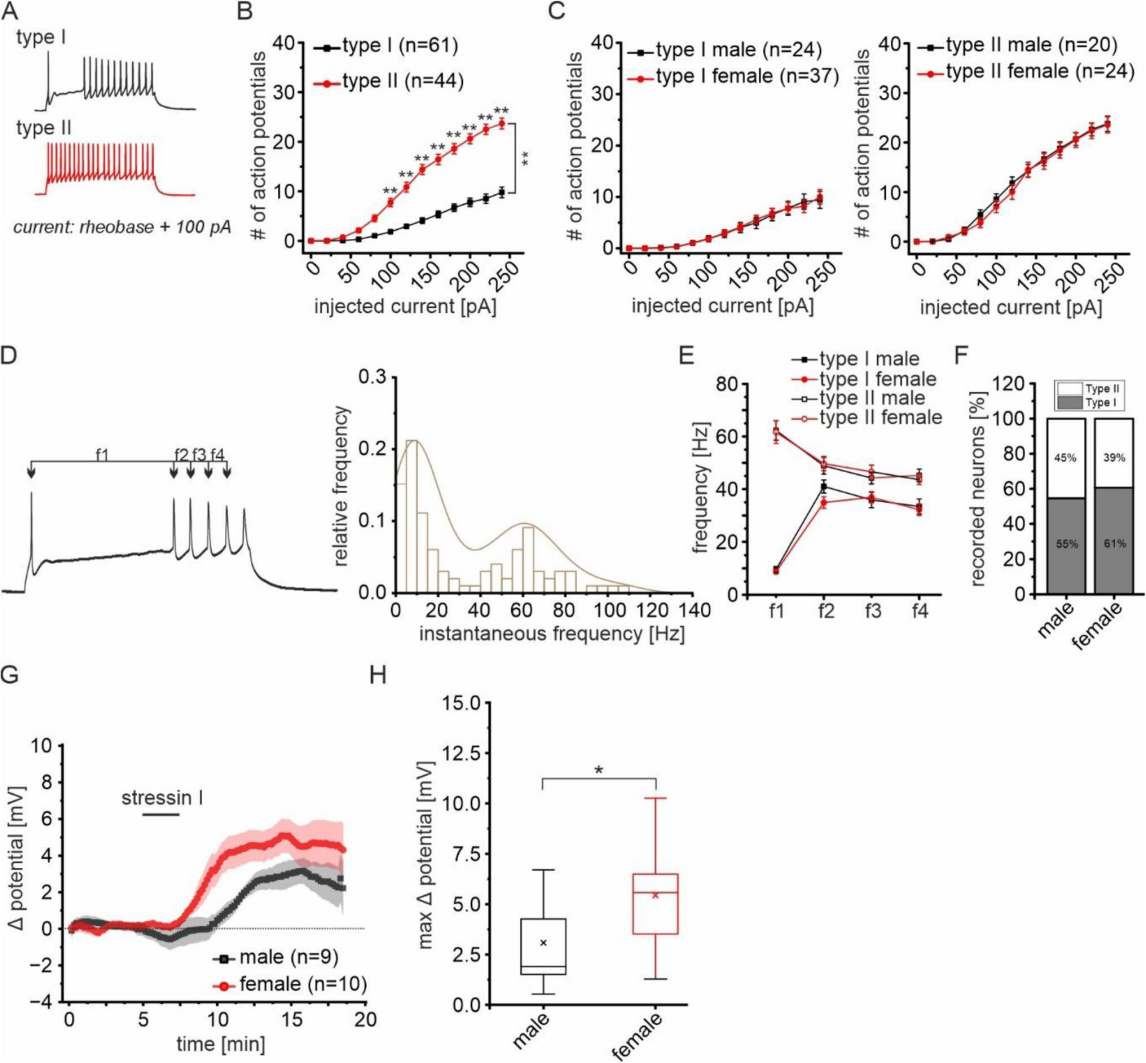
Comparison of type I and type II NPS neuronal properties between male and free-cycling female mice. **A**) Examples of action potential generation in type I and type II neurons in response to + 100 pA current injections recorded in current clamp mode at a membrane potential of −80 mV. **B**) Input–output relationship (number of generated action potentials vs. injected current; current duration 500 ms) recorded from type I and type II NPS neurons. **C**) Comparison of discharge patterns of type I and type II neurons separated according to sex. No significant differences between sexes were detected. **D**) Example of action potential generation of a type I neuron and marking of the first 4 inter-spike intervals used to calculated instantaneous frequencies (f1 to f4). The plot of all recorded first instantaneous frequencies (f1; type I and type II from males and females) shows a bimodal distribution. **E**) Quantification of f1 to f4 did not yield significant differences between sexes. **F**) Percentages of recorded type I and II neurons from male and female mice. While type I neurons appear slightly more abundant, there are no sex-dependent differences. **G**) Average time course of the membrane potential changes in NPS neurons of male and female mice in response to 500 nM stressin I applications. **H**) Quantification of the maximal change of the membrane potential following stressin I application in NPS neurons from male and female mice

In the next set of experiments, we performed whole-cell current-clamp recordings from NPS neurons in the periLC region in acute slice preparations from male and female mice. We analyzed in total 61 type I neurons and 44 type II neurons (Fig. 3A). We analyzed the number of generated action potentials in response to depolarizing current injections from a membrane potential of −80 mV (pulse duration 500 ms; ΔI: + 20 pA/sweep). Analysis revealed that type II neurons generate significantly more action potentials in response to current injections between + 100 and + 240 pA as compared to type I neurons (RM ANOVA: interaction cell type x current injection: F(11,1122) = 59.31; *p* < 0.0001; Fig. 3B). In a next step, we separated the recorded neurons according to the sex of the mice, comparing the discharge pattern of type I and type II neurons from male and female mice (Fig. 3C). We did not find any differences regarding the number of generated action potentials in type I or type II neurons from males and females. One hallmark of the type I neurons is the delay between the first generated action potential, which is usually faster generated than the activation of the I_A_-current, and the consecutive generated action potentials (Fig. 3D). We analyzed the instantaneous frequencies (f1 to f4) between the first 5 generated action potentials in type I and type II neurons. Plotting the distribution of the first instantaneous frequency (f1) for type I and type II neurons shows a two-peaked distribution over the frequencies, where the relative frequency of type I neurons peaked around 8.9 Hz and for type II neurons around 61 Hz. Although f1 clearly distinguishes type I from type II neurons, no differences regarding the frequencies of f1 to f4 were found between neurons recorded from males and females (Fig. 3E). Of note, the abundance of type I and type II neurons recorded from males and females was not significantly different (Chi squared test: χ^2^: 0.392; *p* = 0.53; Fig. 3F).

Additionally, we compared action potential properties generated in both NPS neuronal subpopulations recorded from male and female mice (Table 1). Parameters were analyzed using a Two-Way ANOVA with sex and cell type as factors. Overall, type II NPS neurons generate the action potentials at a slightly more negative threshold potential (threshold type II: males: −38.82 ± 1.16 mV; females: −39.19 ± 0.9 mV; threshold type I neurons: males: −35.44 ± 1.04 mV; females: −36 ± 0.73; Table 1) and generate action potentials with significantly larger peak amplitude (AP amplitude type II neurons: males: 66.5 ± 1.18 mV; females: 66.42 ± 0.85 mV; type I neurons: males 60.6 ± 1.18 mV; females: 58.81 ± 1.21 mV; Table 1). Additionally, action potentials of type I neurons display slower kinetics in recordings from both sexes (rise time, decay time and half-width; results see Table 1). These data underscore the presence of NPS-expressing subpopulations in the periLC region.

**Table 1:** Active membrane properties od type I and type II neurons recorded from male and female mice. All values are mean ± S.E.M.; presented p-values are from multifactorial ANOVA including sex and cell type

	female		male		
	Type I	Type II	Type I	Type II	*p*-value
AP amplitude [mV]	58.81 ± 1.21	66.42 ± 0.85	60.60 ± 1.53	66.50 ± 1.18	*main effect cell type*: 0.000001
threshold [mV]	−36.02 ± 0.73	−39.18 ± 0.90	−35.44 ± 1.04	−38.82 ± 1.16	*main effect cell type*: 0.0009
rise time [ms]	0.55 ± 0.02	0.39 ± 0.01	0.46 ± 0.02	0.36 ± 0.01	*main effect cell type*: 0.000000005 main effect sex: 0.0104
decay time [ms]	1.33 ± 0.04	1.08 ± 0.03	1.33 ± 0.04	1.16 ± 0.04	*main effect cell type*: 0.0000013
half-width [ms]	1.17 ± 0.04	0.92 ± 0.03	1.12 ± 0.04	0.98 ± 0.03	*main effect cell type*: 0.0000005
fAHP [mV]	−17.17 ± 0.47	−18.22 ± 0.60	−16.38 ± 0.53	−14.88 ± 0.60	*interaction sex x cell type*: 0.0243
mAHP [mV]	−13.96 ± 0.54	−16.56 ± 0.66	−13.62 ± 0.57	−13.44 ± 0.60	*interaction sex x cell type*: 0.025

In our previous work we have shown that NPS-expressing neurons in the periLC express functional CRF1 receptors, and that CRF1 activation depolarizes NPS neurons [11]. To identify possible sex-specific differences regarding CRF1-induced membrane depolarizations, we applied 500 nM stressin I for two minutes during current clamp recordings at −60 mV of NPS neurons in brain slices from male or female mice. Stressin I depolarized NPS neurons in slices from both sexes, apparently inducing a depolarization with earlier onset in females (Fig. 3G). Analyzing the maximal stressin I-induced membrane depolarization, we found that the average depolarization in females was 5.4 ± 0.78 mV and 3.08 ± 0.73 mV in males (Fig. 3H). The difference observed between females and males was significant (t-test: t = −2.203,DF = 16.99; *p* = 0.042; males *n* = 9; females *n* = 10). These data indicate that CRF activates NPS neurons in both sexes, with higher impact in females.

### Glutamatergic and GABAergic spontaneous synaptic transmission on NPS neurons

**Fig. 4:**
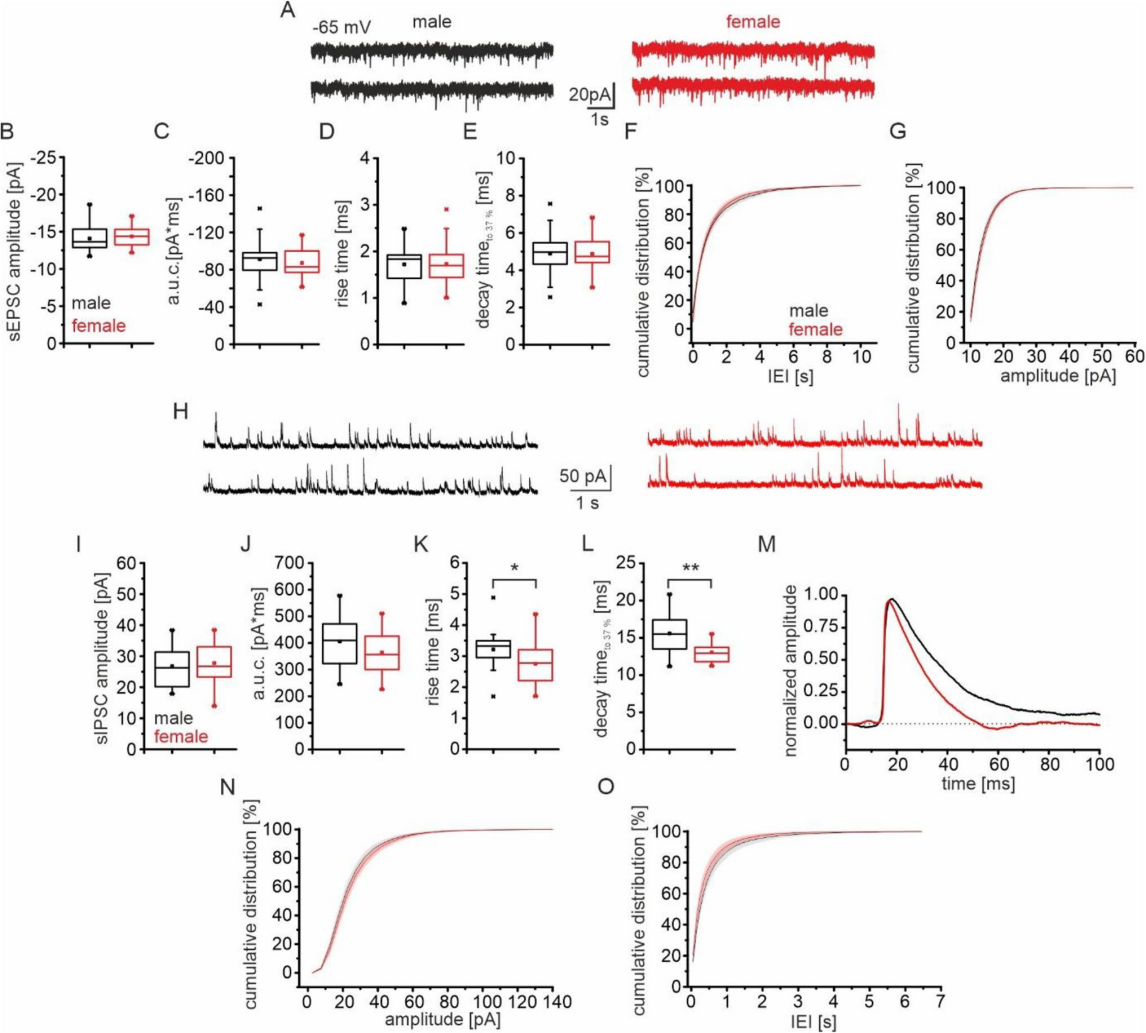
Glutamatergic and GABAergic spontaneous synaptic transmission on NPS neurons recorded from male and female brain slices. **A**) Example traces of spontaneous excitatory postsynaptic currents (sEPSCs) recorded at −65 mV holding potential in voltage-clamp mode in identified NPS neurons in brain slices from males (black) and females (red). **B**) sEPSC amplitude (male *n* = 28; female *n* = 35). **C**) Area under the curve (a.u.c.). **D**) Rise time and **E**) decay time contant. **F**) Cumulative distribution of the inter-event interval. **G**) Cumulative distribution of the sEPSC amplitude. **H**) Example traces of GABAergic sIPSCs recorded at −5 mV holding potential in acute brain slices from male (black) and female (red) mice. I) sIPSC amplitude (male *n* = 17; female *n* = 19). **I**) sIPSC amplitude, a.u.c. (**J**), rise time (**K**), and decay time constant (**L**). **M**) Example of averaged and amplitude-normalized sIPSCs derived from recordings in male (black) and female (red) slices. **N**) and **O**) Cumulative distribution of inter-event intervals and amplitudes, respectively

In a next set of experiments, we addressed the question, if excitatory glutamatergic and inhibitory GABAergic synaptic transmission onto NPS neurons is different in males and females. We recorded spontaneous excitatory postsynaptic currents (sEPSCs) in the whole-cell voltage-clamp mode at a holding potential of −65 mV to electrotonically isolate sEPSCs from spontaneous inhibitory postsynaptic currents (sIPSCs) in absence of pharmacological inhibition to leave network activity intact (Fig. 4A). We recorded in 28 identified NPS-eGFP positive neurons in males and 35 in females. Analyzing the sEPSCs, we did not find any statistically significant differences between recordings from male and female mice regarding mean amplitude (males: −15.6 ± 0.7 pA; females: −16.1 ± 0.7 pA; Fig. 4B), area under the curve (males: −91.2 ± 3.7 pA*ms; females: −85.9 ± 2.8 pA*ms; Fig. 4C), rise time (males: 1.7 ± 0.08 ms; females: 1.8 ± 0.08; Fig. 4D), or the decay time constant derived from a mono-exponential fit (males 4.8 ± 0.2 ms; females: 4.8 ± 0.2 ms; Fig. 4E). In addition, neither the cumulative distribution of the sEPSC inter-event interval (IEI) nor the cumulative distribution of the amplitudes were different between male and female mice (Fig. 4F and G). These data indicate that on the level of spontaneous synaptic transmission no sex-dependent differences were detected in the excitatory input.

Next, we analyzed recordings from NPS neurons done at a holding potential of −5 mV to electrotonically isolate spontaneous GABAergic inhibitory postsynaptic currents (sIPSCs; Fig. 4H). We recorded 17 NPS neurons from male mice and 19 neurons from female mice. Analyzing sIPSCs, we did not detect differences between males and females regarding mean amplitude of sIPSCs (males: 26.8 ± 1.7 pA; females: 27.8 ± 1.5 pA; Fig. 4I) or a.u.c. (males: 405.7 ± 26.3 pA*ms; females: 364 ± 17.9 pA*ms; Fig. 4J). In contrast, we found sex-dependent differences regarding sIPSC kinetics. In males, the rise time was significantly larger than in females (males: 3.2 ± 0.16 ms; females: 2.7 ± 0.15 ms; t-test: t-value: 2.144, df: 34; *p* = 0.039; Fig. 4K). Moreover, sIPSCs from males showed a slower decay time as indicated by the time constant (males: 15.6 ± 0.66 ms; females: 13.1 ± 0.3 ms; t-test: t-value: 3.621, df: 34, *p* = 0.002; Fig. 4L and M). In contrast, we did not find any significant differences between males and females regarding the cumulative distribution of sIPSC inter-event interval or amplitude (Fig. 4N and O). These data indicate, that there are sex-dependent differences between sIPSC kinetics between males and females, where males display slower kinetics of the recorded sIPSCs.

### Influence of female estrous cycle on neuronal action potential generation

**Fig. 5:**
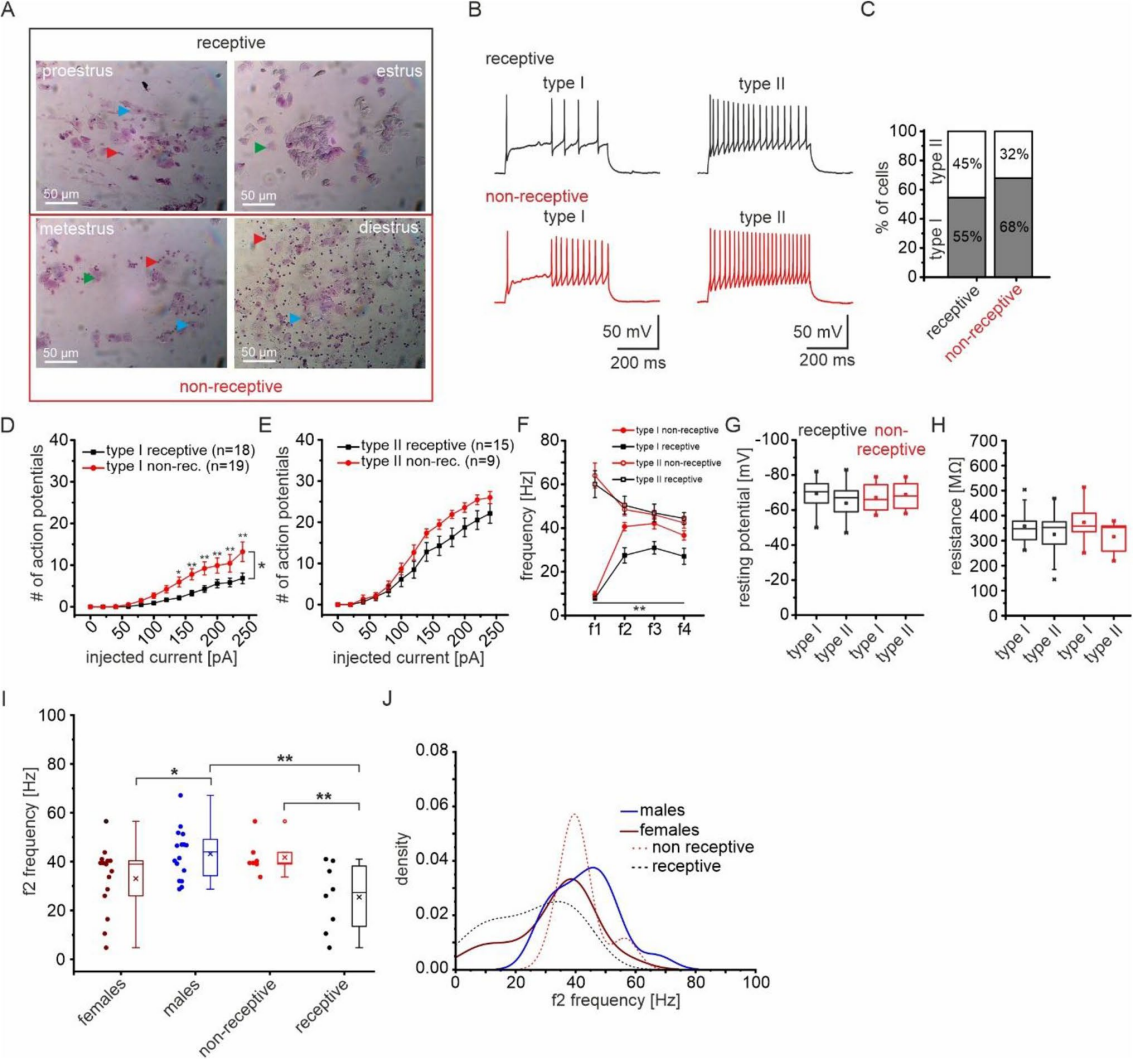
Influence of the receptive stage of female mice on electrophysiological properties of type I and type II NPS neurons. **A**) Examples of post-mortem identification of the estrous cycle by vaginal swab histology. Proestrus, estrus, metestrus and diestrus were identified by presence, abundance or absence of leukocytes (red arrow), cornified epithelial cells (green arrow) and nucleated epithelial cells (blue arrow). Proestrus and estrus stages of the cycle were pooled as receptive and metestrus and diestrus as non-receptive stage of the reproductive cycle. **B**) Example traces of type I and type II neurons recorded in current clamp mode in brain slices from receptive (black) and non-receptive (red) females. **C**) Percentages of type I and type II neurons in receptive and non-receptive females. **D**) and **E**) Quantification of input–output relationships in type I and type II neurons depending on receptive stage. Type I neurons show a significant increase in generated action potentials during non-receptive stages. **F**) Analysis of early instantaneous frequencies (f1 to f4) reveals a main effect of the receptive stage in type I, but not type II neurons. **G**) and **H**) Neither the resting membrane potential nor the input resistance of type I and type II neurons was affected by the receptive stage. **I)** Analysis of the f2-frequency in males, females, females in the receptive stage and the non-receptive stage. Recorded neurons per mouse were averaged, each data point represents the mean f2 of an individual mouse. **J**) Distribution of the f2-frequency in males, females and receptive and non-receptive females. Data derived from (**I**)

We analyzed neuronal discharge patterns from NPS expressing type I and type II neurons and grouped them according to the estrous cycle of female mice. Estrous cycle stage was determined by vaginal cytology, based on the relative presence of nucleated epithelial cells**,** cornified (keratinized) epithelial cells**,** and neutrophils**.** Proestrus was defined by many nucleated epithelial cells**,** few cornified epithelial cells**,** and no neutrophils**.** Estrus was assigned when most cells were cornified; however, early estrus could still show a small number of nucleated epithelial cells**,** and late estrus could include some neutrophils**.** During metestrus**,** the number of neutrophils increased**.** Diestrus was characterized by many neutrophils and nucleated epithelial cells**,** while the amount of cornified epithelial cells decreased [5], Fig. 5A). We grouped the cycle stages in the receptive stages (proestrus and estrus, black) and the non-receptive stages (metestrus and diestrus, red), which is the more progesterone-associated phase of the cycle. During both stages, receptive and non-receptive, we found both neuronal discharge patterns, delayed and regular firing, in NPS expressing neurons (Fig. 5B). Although there are some changes in percentual distribution of type I and type II neurons recorded during receptive and non-receptive stages, we did not find any statistical significance using the chi squared test (χ^2^ = 1.125, DF = 1, *p* = 0.289; Fig. 5C). In a next step, we analyzed the number of generated action potentials elicited in current clamp by increasing depolarizing current injections (500 ms duration; from 0 to 240 pA; Δ + 20 pA) from a membrane potential of −80 mV. The comparison of type I delayed firing NPS neurons recorded either during the receptive or non-receptive cycle phase revealed a significant difference. Neurons recorded during non-receptive stages displayed a steeper input–output relationship as compared to neurons from the receptive stage (RM ANOVA: significant interaction stage x current injection, F(10,250) = 3.28; *p* = 0.0005; Fig. 5D). In contrast, we did not find a statistical significant interaction of reproductive stages and discharge pattern in recordings of regular firing type II NPS expressing neurons (RM ANOVA: F(10,220) = 1.286; *p* = 0.24; Fig. 5E). When comparing the instantaneous frequencies (f1 to f4) between the first generated action potentials in type I neurons, we found a significant main effect of reproductive stages, indicating that type I neurons during the non-receptive stages display on average higher instantaneous frequencies (RM ANOVA: main effect reproductive stage; F(1,26) = 11.71; *p* = 0.0021; Fig. 5F). The reproductive stages neither had an effect on instantaneous frequencies in type II NPS expressing neurons, nor on the resting membrane potential or input resistance of the recorded type I and type II neurons (Fig. 5G and H). Next, we compared differences in the f2-frequency between males and females on individual mouse level. We averaged all recordings derived from single mice irrespective of the neuronal type (type I or II) and plotted these data for all females, males and for females separated according to the receptive stage (Fig. 5I). Using a OneWay-ANOVA, we found significant differences between males and females, between males and females in the receptive stage of the cycle, and between females in receptive and non-receptive stages (ANOVA: F_3,45_ = 4.94; *p* = 0.005; post hoc test: males vs. females: *p* = 0.02; males vs. receptive females: *p* = 0.0012; receptive vs. non-receptive females: *p* = 0.011; Fig. 5I). We also plotted the distribution of the f2-frequency within the four groups (females, males, receptive and non-receptive females; Fig. 5J). The f2-frequency distribution peaks around 40 Hz in females and 45 Hz in males. Interestingly, non-receptive females display a sharp peak around 40 Hz, whereas receptive females display a flattened distribution around 35 Hz with a broad shoulder towards lower frequencies. Although the distribution in males is less sharp as compared to non-receptive females, the broad extension towards lower frequencies is largely missing. These data indicate that in females, the distribution of discharge frequencies, i.e. these of the initial generated action potentials, is prominently influenced by the receptive stage.

Except for a change in one component of the afterhypopolarizing potential (mAHP, Table 2), we did not find any influence of the reproductive stages on active membrane properties. Notably, the action potential threshold was unchanged by the receptive stages. These data indicate a differential influence on the discharge patterns of type I and type II NPS expressing neurons in the periLC region. Our data point to more increased action potential generation in type I neurons during the non-receptive stages, which is consistent with a reduced I_A_-current.

**Table 2:** Active membrane properties of type I and type II neurons recorded from female mice during receptive and non-receptive stages. All values are mean ± S.E.M.; presented p-values are from multifactorial ANOVA including cycle stage and cell type

	receptive		non-receptive		
	Type I	Type II	Type I	Type II	
AP amplitude [mV]	60.36 ± 1.42	66.62 ± 1.24	57.60 ± 1.82	66.07 ± 0.1	*main effect cell type*: 0.00002
threshold [mV]	−35.96 ± 1.23	−39.21 ± 1.30	−36.06 ± 0.92	−39.13 ± 1.07	*main effect cell type*: 0.012
rise time [ms]	0.53 ± 0.02	0.40 ± 0.02	0.57 ± 0.04	0.36 ± 0.01	*main effect cell type*: 0.012
decay time [ms]	1.34 ± 0.06	1.10 ± 0.04	1.31 ± 0.05	1.06 ± 0.04	*main effect cell type*: 0.00001
half-width [ms]	1.18 ± 0.05	0.94 ± 0.03	1.16 ± 0.05	0.88 ± 0.04	*main effect cell type*: 0.000004
fAHP [mV]	−17.11 ± 0.80	−17.51 ± 0.73	−17.23 ± 0.57	−19.46 ± 0.92	
mAHP [mV]	−13.31 ± 0.79	−15.65 ± 0.78	−14.51 ± 0.74	−18.14 ± 1.01	*main effect cell type*: 0.001 *main effect cycle*: 0.035

## Discussion

The NPS system has been implicated in learning and memory, locomotor activity, fear and anxiety, reward, energy homeostasis, and sleep–wake regulation [31, 37]. Several behavioral studies also point to sex-dependent effects within the NPS/NPSR system, whereas mechanistic information at the level of identified NPS neurons remains limited, with only a few exceptions [8]. The present data therefore provide electrophysiological information that may help to explain sex- and estrous-stage-dependent variation in NPS-related behaviors.

In the periLC region, we identified two electrophysiologically distinct populations of NPS neurons that differ in their mode of action potential generation from relatively hyperpolarized membrane potentials. These populations comprise delayed-onset firing (type I) neurons and regular-spiking (type II) neurons, with type I neurons representing the slightly more abundant class in both sexes. Pharmacological isolation of the transient I_A_ current and its sensitivity to 4-AP indicate that a prominent A-type potassium conductance contributes to the delayed firing phenotype and is most consistent with a major contribution of K_v_-family channels [4]. There are different types of voltage gated potassium channels, that mediate such a transient I_A_ current, e.g. K_v_4, K_v_1.4, K_v_3.3, and K_v_3.4. Since recordings were done in tetraethylammonium (TEA), the observed current is most likely conducted by members of the Kv4 family (i.e. K_v_4.2 and K_v_4.3). Because millimolar 4-AP is not fully subtype-selective, our data do not identify the underlying channel complex definitively, and we cannot rule out the presence of e.g. K_v_1.4 channel subtypes,however, the current kinetics and the firing behavior strongly support a K_v_4-like conductance. Such I_A_ currents activate rapidly during depolarization after recovery from inactivation at hyperpolarized membrane potentials and thereby act as a brake on excitability, delay the first spike, and reduce neuronal gain [4, 16]. Accordingly, the attenuated input–output relationship of type I neurons is well explained by the larger transient outward current in this subgroup. The smaller current amplitude and altered kinetics in type II neurons may reflect lower functional expression of Kv4-containing channels and/or differences in auxiliary subunits such as KChIPs, which can modify channel trafficking and gating [17], [34], [38].

Our data do not support a broad sex difference in intrinsic membrane properties of periLC NPS neurons. Type I and type II neurons were present in similar fractions in males and females, and neither the input–output analysis nor the instantaneous firing frequencies revealed robust baseline sex-specific differences. Likewise, passive membrane properties and most active membrane properties were largely similar between sexes. Any subtle differences in action potential waveform should therefore be interpreted cautiously and not as evidence for a general sex difference in excitability. In addition, we analyzed spontaneous excitatory and inhibitory synaptic transmission on NPS neurons in brain slices of male and female mice. The degree of excitation and inhibition can shape the activity pattern of NPS neurons. Sex-dependent differences in synaptic transmission have been reported in a number of scientific publications analyzing either spontaneous and/or miniature synaptic transmission [22, 29]. Here, we do not find sex-dependent differences regarding sEPSC or sIPSC amplitude, a.u.c. or IEIs, but we detected sex-dependent differences regarding sIPSC kinetics, where sIPSCs of male mice showed slower kinetics. Similar differences in kinetics haven been reported in neurons of the central amygdala in rats, where GABAergic transmission had faster postsynaptic kinetics in females [18]. Differences in synaptic transmission per se and also postsynaptic current kinetics might well influence the pattern of neuronal activity. Of note, we did not record miniature postsynaptic currents, which limits the interpretation of the data presented here. In the present work, we extended our earlier findings that CRF1 activation induces membrane depolarizations in NPS neurons [11] by showing that stressin I application induces significantly stronger membrane depolarizations in females as in males. As a functional consequence one might speculate that during stress and concomitant CRF release, periLC NPS neurons in females might be activated more effectively.

Assigning recordings from female mice to receptive and non-receptive stages of the estrous cycle revealed differences primarily in type I neurons. During non-receptive stages, type I NPS neurons generated more action potentials and displayed higher instantaneous frequencies, whereas type II neurons showed no statistically significant change. Because estrous stages were pooled into receptive (proestrus/estrus) and non-receptive (metestrus/diestrus) groups, and because vaginal cytology is only an indirect proxy for circulating hormone concentrations, these findings should be interpreted as estrous-stage-associated rather than as a direct demonstration of estradiol action [5], [6]. Nevertheless, the most parsimonious explanation is that the conductance responsible for delayed firing in type I neurons is reduced during non-receptive stages. A smaller I_A_ current would shorten the delay to first spike and increase firing frequency, which matches our physiological observations. In male NPS neurons, when averaged per mouse irrespective of the neuronal discharge type, higher early f2 firing frequencies were detected than in females overall. These early firing frequencies are shifted towards lower frequencies in the total female sample, most likely driven by a strong shift towards low f2-frequencies predominantly in receptive females. These findings support the idea that the estrous cycle impacts electrophysiological properties. While the distribution in males seems to be more homogenous, the data do not rule out a hormonal influence on discharge properties in males.

Importantly, we did not detect corresponding changes in passive properties or spike threshold that would strongly support an alternative explanation. At the same time, this mechanistic interpretation remains provisional because I_A_ was not measured directly across estrous stages. The literature on hormone-dependent regulation of Kv4 channels is heterogeneous and likely depends on cell type, brain region, and species. Still, data from corticotropin-releasing hormone neurons in the paraventricular hypothalamus show that estradiol can increase I_A_ current density and thereby shape excitability [28]. It is therefore plausible that especially type I periLC NPS neurons regulate functional expression or modulation of K_v_4-like channels across the estrous cycle. In addition, other modulatory systems present in periLC NPS neurons, including dynorphin/KOR and PACAP/PAC1 signaling, could influence Kv4-dependent excitability and may contribute to cycle-dependent effects [13], [23].

Brainstem NPS neurons are recruited by stress-related corticotropin-releasing factor (CRF) signaling [11]. This point is relevant for the present findings because estrous-stage-dependent changes in intrinsic excitability could alter how strongly periLC NPS neurons respond to stress-related synaptic and neuromodulatory input. Thus, ovarian-state-dependent tuning of intrinsic membrane properties may interact with previously described CRF sensitivity to shape the recruitment of the NPS system under challenging conditions. One limitation of the present work is the lack of electrophysiological in vivo data, which could provide direct evidence for a possible cycle-dependent transition of activity patterns of identified neurons. Although these kinds of experiments are beyond the scope of this work presented here, future research should examine the influence of cycle-stages on the NPS system in more detail.

Behavioral state differences across the estrous cycle have been documented for social interaction, motor learning, locomotion, and reward-related behaviors [1, 6, 15]. The NPS system has been implicated in many of these functions [31, 37]. Recent anatomical and functional studies further indicate that NPS neurons in and around the LC form part of a broader parabrachial/peri-coerulear cluster involved in arousal and breathing control [2]. Candidate projection targets of periLC NPS neurons, including the BNST, lateral hypothalamic area, paraventricular thalamus, and posterior subthalamic region, are well positioned to link internal state, stress responsiveness, autonomic/endocrine output, and motivated behavior [2]. Consequently, estrous-stage-dependent changes in spike generation and input–output transformation of periLC NPS neurons may alter signal integration and output to these circuits. Future experiments should therefore determine whether the cycle-dependent physiological changes described here are accompanied by direct changes in IA, by hormone-dependent modulation of upstream afferent systems, and by altered behavioral recruitment of defined NPS projection pathways. A more detailed understanding of how sex hormones shape peptidergic brainstem circuits will be important for interpreting sex differences under both physiological and pathophysiological conditions.

## Data Availability

Data recorded and analyzed for this manuscript are available upon request.
